# Proposal of Major Environmental Impact Categories of Construction Materials Based on Life Cycle Impact Assessments

**DOI:** 10.3390/ma15145047

**Published:** 2022-07-20

**Authors:** Hyeong-Jae Jang, Yong-Han Ahn, Sung-Ho Tae

**Affiliations:** 1 Department of Architectural Engineering, Hanyang University, 55 Hanyangdaehak-ro, Sangrok-gu, Ansan 15588, Korea; duethj@gmail.com; 2 School of Architecture & Architectural Engineering, Hanyang University, 55 Hanyangdaehak-ro, Sangrok-gu, Ansan 15588, Korea

**Keywords:** construction materials, environmental impact category, life cycle impact assessment

## Abstract

The “Korean New Deal” policy in South Korea emphasizes the necessity of a substantial and timely response to global climate change. In addition to carbon emissions, construction materials have various environmental impacts that necessitate serious considerations. Therefore, this study aimed to identify the major environmental impact categories of construction materials that reflect their diverse environmental impact characteristics using life cycle assessment. To this end, eight environmental impact categories were assessed for seven major construction materials. The contributions of all construction materials to these environmental impact categories were then analyzed to derive major environmental impact categories with contributions ≥95% or higher for each construction material. Consequently, global warming potential and abiotic depletion potential were derived as major environmental impact categories for all seven construction materials. In the case of ready-mixed concrete and cement, the photochemical oxidant creation potential was also found to be an environmental impact category that needs to be considered further. Thus, a study that defines environmental impacts must be considered in conjunction with the carbon emissions of building materials, and presenting the criteria for evaluating the defined environmental impacts is essential.

## 1. Introduction

The “Korean New Deal”, announced as a goal in response to global climate change policy, was introduced in South Korea in April 2020 as a large-scale national project for innovative growth in the post-Coronavirus disease (COVID-19) era. Severe environmental problems have led to efforts to reduce carbon emissions as carbon neutrality has recently become an area of focus [1,2]. In addition to the increase in carbon emissions, global warming is caused by various other environmental impacts [3,4,5]. Carbon dioxide (CO_2_), methane (CH_4_), nitrous oxide (N_2_O), hydrofluorocarbons (HFCs), perfluorocarbons (PFCs), and sulfur hexafluoride (SF_6_) have been designated as the six major greenhouse gases (GHGs) that cause global warming at the third Conference of the Parties for the United Nations Framework Convention on Climate Change [6,7,8]. Further, the atmospheric concentrations of CH_4_ and N_2_O have steadily increased over the past 30 years, causing air quality deterioration [9,10]. Thus, the comprehensive long-term monitoring of carbon, nitrogen, and sulfur compounds is required in terms of air quality and response to climate change [11]. For example, concern over ozone depletion led to the formulation of the Montreal Protocol on substances that deplete the ozone layer in 1987. Accordingly, this protocol provided the regulations for producing and using chlorofluorocarbons (CFCs) [12]. Substances, such as SO_2_ and NO_x_, are known to result in acidification and have been specified as pollutants affecting human health. Therefore, their emissions have been subjected to restrictions for atmospheric environment management [13]. The Korean Government has also operated a system to assess various environmental impacts, such as global warming, acidification, eutrophication, and resource consumption, for industrial products [14]. However, only a limited number of construction materials have achieved certification; additionally, studies on various environmental impacts of construction materials are also insufficient [15,16]. In particular, given that studies on the assessment of environmental impacts of construction materials have been focused on evaluating carbon dioxide emissions that affect global warming, research is required on various environmental impacts of the production and use of construction materials on the global environment [17,18].

Using life cycle assessment (LCA), the present study attempts to identify the major environmental impact categories of construction materials that reflect their differences in environmental impact characteristics. To this end, in this study, seven major construction materials were selected and their environmental impacts were assessed. For each construction material, the following eight environmental impacts were assessed: global warming, resource consumption, photochemical oxidation, acidification, ozone depletion, eutrophication, eco-toxicity, and human toxicity. The contribution of each construction material to the eight environmental impact categories was analyzed, and major categories of environmental impact were derived for each building material with a contribution of approximately more than 95%.

Research is needed on environmental impacts of the production and use of construction materials on the global environment, given that studies have examined carbon dioxide emissions that affect global warming as part of the assessment of environmental impacts of construction materials.

**Figure 1:**
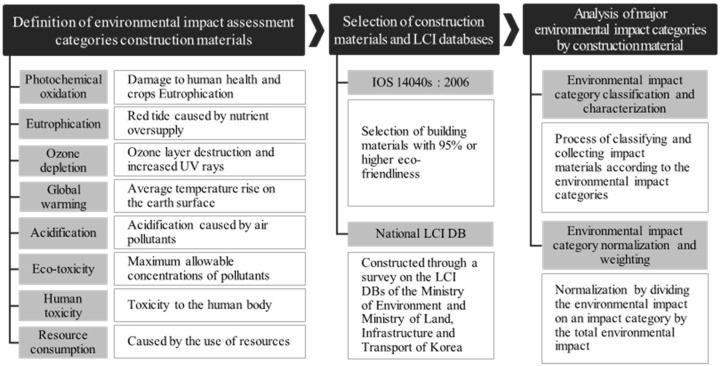
Research flow and framework.

In addition, the purpose of this study is to propose major environmental impact categories of building materials using life cycle impact assessment (LCIA) based on the research flow, as shown in Figure 1.

## 2. Literature Review

### 2.1. Review of Research Direction

In this section, the existing literature and limitations are examined to set the direction of research.

Ref. [18] compared three different LCIA methods and found important differences while evaluating aquatic ecotoxicity (EDIP 97, CML 2001, and Impact 2002+) among them. Ref. [19] analyzed major building tasks and materials in order to assess the environmental impact of apartment buildings in Korea. In this study, six major building materials—ready-mixed concrete, rebar, insulation materials, concrete bricks, glass, and gypsum boards—were identified, accounting for over 95% of the values of six environmental impact categories.

Refs. [20,21] compared nine different LCIA methods to assess the impacts of metals on humans to assess the impacts on health, aquatic, and terrestrial ecosystems. They found differences among all methods in the characterization factor (CF) and the calculation method used. In the case of impacts on aquatic and terrestrial ecosystems, the characterization step was identified as crucial. Ref. [22] evaluated the CO_2_ emission reduction rates over the life cycle of long-life apartment houses (Types II and III) using high-durability and maintenance technology from general apartment houses (Type I) as a reference, finding that the maximum CO_2_ emission reduction rates of long-life apartment houses were 36.18% and 33.04%, respectively. Ref. [23] analyzed different cases of biofuels. They compared seven methods and analyzed the results that varied owing to the difference in the selected factors for global impact.

Ref. [24] analyzed the major building materials in terms of environmental impact evaluation of school buildings in South Korea, with three existing school buildings selected as the analysis targets. Building materials were analyzed in terms of cumulative weight and six environmental impact categories (global warming potential, abiotic depletion potential, acidification potential, eutrophication potential, ozone-layer depletion potential, and photochemical oxidation potential). Ref. [25] conducted the correlation analysis for a construction material dataset and reported the problem associated with the fact that the LCA results can be different depending on the selection of impact factors by the LCIA professional. They presented guidelines for selecting environmental impact factors with high reliability based on case analysis.

These studies described LCIA methodologies, analyzed them following their evaluations, and determined the problems based on identifying the differences among them. However, they had limitations in revealing the benefits and shortcomings of the methodologies. Recent studies found that results may vary depending on the selection of impact factors by the person responsible for applying the LCIA technique and attempted to present guidelines on selecting more reliable factors based on case analysis.

As these studies only revealed the limitations of LCIA methodologies or selected environmental impact factors by analyzing a small sample containing special cases, the selection and weighting of environmental impact categories for LCA dedicated to building materials in the construction field have not been performed.

Thus, a study that defines the environmental impacts must be considered together with the carbon emissions of building materials, and presenting the criteria for evaluating the defined environmental impacts is essential. Accordingly, an attempt was made to derive the environmental impact categories with contributions ≥95% that reflect the contributions of major construction materials, and to propose environmental impact categories specialized for each construction material.

### 2.2. Review of LCIA Methodologies

In this section, the existing LCIA methodologies are examined to define the environmental impact categories suitable for building materials. Considering the environmental performance of a product can be derived differently depending on the environmental impact assessment category or assessment criterion. Determining appropriate impact categories and assessment criteria in accordance with the assessment objective and purpose is essential. Although most methodologies have been developed primarily in Europe, various LCIA methodologies have been developed by many researchers over time. Each LCIA methodology defines environmental impact categories and evaluation methods for each category. LCIA methodologies are primarily divided into midpoint and endpoint impact categories [26,27,28].

A midpoint category consists of environmental impacts related to inputs and outputs and identifies specific problems, such as climate change, ozone depletion, acidification, and fossil fuel depletion. It is also referred to as a problem-oriented approach, and it quantitatively assesses the degree of environmental problems caused by pollutant emissions from environmental mechanisms. Representative methodologies, such as CML 2001, EDIP 2003, and TRACI CML 2001, were developed by the Center of Environmental Science of Leiden University to assess the environmental impact categories using Ecoinvent [29]. These are internationally accepted methodologies that provide normalization factors for Europe and the rest of the world. EDIP 2003 is an improvement of the EDIP 97 methodology developed by the Technical University of Denmark in mid-1997. It defines eight environmental impact categories for Europe, except for global warming and ozone depletion, which consider the earth as the reference area. Tool for the Reduction and Assessment of Chemical and Other Environmental Impacts (TRACI), an environmental impact assessment tool developed by the United States Environmental Protection Agency (US EPA) in 2003, assesses nine categories of environmental impacts [30]. The ozone depletion and greenhouse effect sectors were developed at the global level, whereas the other sectors were developed based on data from North America. TRACI does not include the assessment of resource depletion [31].

Conversely, the endpoint level methodology is focused on the final damage caused by environmental loads: the environmental impact categories cover damages to human health, ecosystem diversity, and resource availability, as well as the destruction of the ecosystem. The endpoint impact–based methodology is also referred to as the damage-oriented approach: representative methodologies include the Eco-indicator 99 and EPS 2000 [32]. The Eco-indicator 99, developed in 1999, presents the assessment results for resources, ecosystem quality, and human health items. It defines the degrees of influence of inputs or outputs on each item to obtain the damage estimation for each of the three impact categories. In addition to assessing these three items, normalization and weighting factors are defined by classifying humans from three perspectives. In an effort to present environmental loads as costs, EPS 2000 methodology was developed between 1990 and 1991. An analysis of the impacts of emissions on each category of environmental impact is presented, as is an evaluation of the importance of each category of environmental impact. Furthermore, EPS 2000 defines five environmental load categories: human health, production capacity of the ecosystem, abiotic resources, impact on biodiversity, and cultural and recreational values. Table 1 summarizes each LCIA methodology [33].

Eco-indicator 99 and EPS 2000 possess different benefits and shortcomings owing to the differences in their methodological characteristics. Although the midpoint impact category-based methodology generally includes all environmental impacts, it is difficult to understand the assessment results [34,35,36,37]. On the contrary, in the case of the endpoint impact-based approach, the assessment results can be easily understood. However, it cannot be stated that the assessment considers all the losses and impact factors caused by different environmental impacts. The characteristics of these LCIA methodologies are diverse, and inevitably, the result of each LCIA methodology is significantly different from another depending on the selection of impact factors. Nevertheless, there is no internationally approved guideline according to the requirements of the International Standardization Organization (ISO) [22]. Specifically, in the construction sector that accounts for 38% of the domestic carbon emissions, selecting impact factors and defining assessment scopes for them is necessary for LCA.

Therefore, in this study, the impact categories and assessment criteria have been applied based on the universally applicable CML 2001 methodology by providing global impact to facilitate the clear specification of the environmental impact factors and their scope.

## 3. Category Definition for Assessing the Environmental Impacts of Construction Materials

### 3.1. Overview

**Figure 2:**
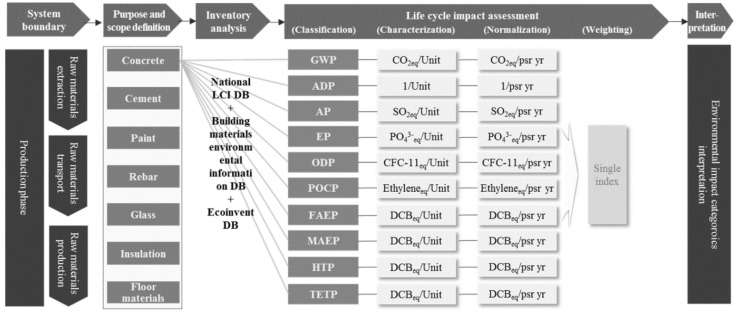
Concepts of the midpoint and endpoint impact–based models.

As shown in Figure 2, this study is conceptually based upon a theoretical examination of life cycle assessment (LCA) and its impact categories. A database (DB) of the environmental impact specialization values of construction materials for each impact category is required for the LCA of a building. Building owners and architects can easily determine the environmental impact of a building by multiplying the quantities of major construction materials with their environmental impact specialization values. The objective of this study is to estimate environmental impact specialization values from the LCA database of a building.

The purpose of this study is to identify the major environmental impact categories for each construction material in order to reflect its characteristics through life cycle assessment. This study focused on impact assessment, which is the third of the four stages of LCA. The specializations and weightings specified in ISO 14044:2011 were employed. We have used the eight categories of the CML 2001 methodology to derive environmental influence factors that should be considered in addition to CO_2_ emissions in the construction industry.

### 3.2. Environmental Impact Categories of Construction Materials

#### 3.2.1. Global Warming Potential

The phenomenon of global warming is the rise in the average temperature of the Earth’s surface resulting from the emissions of greenhouse gases, such as carbon dioxide. In terms of global warming, CO_2_ is the reference substance. A total of 23 substances have an impact, including CH_4_, N_2_O, HFCs, and SF_6_. In this study, global warming was calculated using CO_2_ as the reference substance and the global warming potential (GWP) provided by the Intergovernmental Panel on Climate Change (IPCC) applied to the environmental loads of the impact substances as follows [39],

(1) $$\mathrm{Global}\;\mathrm{warming}=\sum Load_{\left(i\right)}\times GWP_{\left(i\right)},$$

where $Load_{\left(i\right)}$ and $GWP_{\left(i\right)}$ are the environmental load and global warming potential of the impact substance i, respectively.

#### 3.2.2. Ozone Depletion Potential

Ozone depletion is defined as the reduction in density of the ozone layer in the stratosphere at distances in the range of 15–30 km above the ground, leading to skin cancer in humans, primarily caused by CFCs, because of the increase in ultraviolet rays reaching the Earth’s surface. In relation to ozone depletion, trichlorofluoroethylene (CFC-11) is the reference substance, and there are 22 impact substances, including bromotrifluoroethylene (Halons 1301), hydrobromofluorocarbons, hydrochlorofluorocarbons, methyl bromide, and methyl chloride. Based on the ozone depletion potential (ODP) provided by the World Meteorological Organization (WMO) to the environmental loads of the impact substances, the ozone layer impact was calculated using CFC-11 as the reference substance [40,41].

(2) $$\mathrm{Ozone}\;\mathrm{layer}\;\mathrm{impact}=\sum Load_{\left(i\right)}\times ODP_{\left(i\right)},$$

where $Load_{\left(i\right)}$ and $ODP_{\left(i\right)}$ are the environmental load and ozone depletion potential, respectively, of the impact substance i.

#### 3.2.3. Acidification Potential

Acidification is the increase in the acidity of rivers, streams, and soil owing to atmospheric pollutants, such as SO_2_, NH_3_, and NO_x_. It increases the elution of heavy metals and affects ecosystems, such as the nutrient and feed supply of fish, plants, and animals. The reference substance of acidification is SO_2_, with 23 impact substances, including NH_3_, H_2_SO_4_, and NO_x_. Acidification was calculated using SO_2_ as the reference substance. The acidification potential (AP) described by Ref. [42], and EDIP 2003 were applied to the environmental loads of the impact substances as follows,

(3) $$\mathrm{Acidification}=\sum Load_{\left(i\right)}\times AP_{\left(i\right)},$$

where $Load_{\left(i\right)}$ and $AP_{\left(i\right)}$ are the environmental load and acidification potential, respectively, of the impact substance i.

#### 3.2.4. Abiotic Depletion Potential

Abiotic depletion is caused by the prolonged use of natural resources, such as groundwater and fossil fuels. Reference substance is antimony, Sb, and there are over 90 impact substances, including Al, Cd, Fe, Au, Hg, natural gas, and crude oil. Sb was used as the reference substance in calculating abiotic depletion. Abiotic depletion potential (ADP) has been applied to the environmental loads of impact substances by Ref. [43],

(4) $$\mathrm{Abiotic}\;\mathrm{depletion}=\sum Load_{\left(i\right)}\times ADP_{\left(i\right)},$$

where $Load_{\left(i\right)}$ and $ADP_{\left(i\right)}$ are the environmental load and abiotic depletion potential, respectively, of the impact substance i.

#### 3.2.5. Photochemical Oxidant Creation Potential

In photochemical oxidation, pollutants in the air react with sunlight to produce chemical compounds, such as ozone. In addition to adversely affecting ecosystems, these chemicals cause adverse effects on human health and crop growth. As a reference, ethylene is used to create photochemical oxidants, while there are more than 100 impact compounds, including acetone, benzene, CO, ethane, methane, and toluene. Accordingly, photochemical oxidant formation was calculated using ethylene as the reference substance and applying the photochemical oxidant creation potential (POCP) described by Refs. [44,45], to the environmental loads of the impact substances,

(5) $$\mathrm{Photochemical}\;\mathrm{oxidant}\;\mathrm{creation}=\sum Load_{\left(i\right)}\times POCP_{\left(i\right)},$$

where $Load_{\left(i\right)}$ and $POCP_{\left(i\right)}$ are the environmental load and photochemical oxidant creation potential, respectively, of impact substance i.

#### 3.2.6. Eutrophication Potential

Eutrophication occurs when an oversupply of nutrients causes the growth of algae in the aquatic ecosystem, leading to the appearance of red tides. The reference substance for eutrophication is PO_4_^3−^, with 11 impact substances, including NH_3_, NH^+4^, N_2_, NO_2_, and P. Eutrophication was calculated using PO_4_^3−^ as a reference substance, and the eutrophication potential (EP) was applied to the environmental loads of the impact substances as follows,

(6) $$\mathrm{Eutrophication}=\sum Load_{\left(i\right)}\times EP_{\left(i\right)},$$

where $Load_{\left(i\right)}$ and $EP_{\left(i\right)}$ are the environmental load and eutrophication potential, respectively, of impact substance i.

#### 3.2.7. Terrestrial Eco-Toxicity Potential

Eco-toxicity is obtained based on the maximum allowable concentration of pollutants for each toxicity class. To quantify accurately the toxicity of a substance, it is necessary to establish the path and quantify the extent of exposure of each ecosystem to that particular substance. In this study, the initial emissions of toxic substances and their effects on ecosystems and humans were considered. The maximum tolerable concentrations (MTCs) developed by the US EPA were used to quantify toxic substances. For soil toxicity, the MTC was determined by estimating the acute toxicity of soil organisms and the quantitative activity relationship of the structure. The LD50 or EC50 values for all substances were obtained experimentally, and acute toxicity or chronic (no observed effect) levels were predicted according to the molecular structure. When acute toxicity data were used, the lowest value among the toxicological data of soil organisms was selected and multiplied by the safety factors of 0.01 or 0.001, depending on the number of data, to estimate MTC. The specialization coefficient for ecological aquatic toxicity is defined as follows,

(7) $$ECA=X_{a}\times E_{a}=\frac{1}{MTC_{EPA}},$$

where $X_{a}$ is the exposure factor (set to one) and $E_{a}$ is the effect factor (i.e., reciprocal of the MTC).

#### 3.2.8. Particulate Matter Formation

Particulate matter refers to atmospheric pollutants with particle sizes lower than 10 μm, generated during concrete production and dismantling/disposal activities in the construction industry; the particulate matter formation (PMF) factor is used as the reference substance. Impact substances include NH_3_, NO_x_, and SO_2_. PMF is calculated as the value of particulate matter (PM10) by applying the PMF expression described in ReCiPE 2008,

(8) $$Particulate\;Matter\;Formation=\sum_{r=1}^{r=n}\frac{PDI_{r,x,s,e}}{HLV_{r,x}},$$

where *Load*_(*i*)_ is the environmental load of impact substance *i*, classified as a particulate matter substance among the impact categories, and *PMF*_(*i*)_ is the particulate matter formation of the particulate matter impact substance *i*.

## 4. Construction Material and Life Cycle Impact Database Selection

In this study, LCA was performed to analyze the major impact categories of major construction materials. Major construction materials, such as concrete, rebar, paint, glass, cement, insulation, and gypsum board [46], represent over 95% of the environment-friendly building materials exempted by the ISO 14044 standard, and were selected and assessed based on the existing literature. To estimate their environmental impact with high reliability [47], a life cycle impact (LCI) DB of construction materials was created by examining both the national LCI DB (Ministry of Knowledge Economy and Ministry of Environment), constructed according to the direct integration method, and the national DB for building material environmental information (Ministry of Land, Infrastructure and Transport (MOLIT)), as presented in Table 2. For the creation of the detailed LCI DB for construction materials, the national LCI DB was applied preferentially and supplemented by building material environmental information in the national DB. However, the national DB of building material environmental information was applied for shape steel, considering the subdivisions of the LCI DB list. A total of 22 construction materials, i.e., 18 from the national LCI DB and 4 from the national DB for building material environmental information, were examined in detail based on the seven major construction materials.

## 5. Analysis of Major Impact Categories for Each Construction Material

### 5.1. Impact Category Classification for Each Construction Material

In order to collect and classify the data, impact substances were classified and categorized according to the impact categories. The fact-based LCIA methodology in the scientific literature enabled the identification of the influence of each impact material on the global environment. For example, the reference substance for global warming according to the IPCC guidelines is CO_2_, and the impact substances include CFC-11, CFC-114, and CFC-12. The classification results for ready-mixed concrete 25-240-15 using the national LCI DB were 4.20 × 10^2^ kg-CO_2_/m^3^, 2.05 × 10^−9^ kg-CFC-11/m^3^, 2.10 × 10^−9^ kg-CFC-114/m^3^, and 4.40 × 10^−10^ kg-CFC-12/m^3^. Table 3 lists the classification results of the LCI DB of building materials, including ready-mixed concrete 25-240-15, electric-steel deformed bars, and paint–water type.

### 5.2. Impact Category Specialization for Each Construction Material

Impact substances were identified and correlated with the impact categories based on classification. However, there were limitations in the quantitative identification of their impact levels because each impact substance possessed different potentials. The environmental impacts of construction materials can be quantitatively calculated based on specialization, where the emission of each impact substance and its potential by impact category is multiplied and added. CO_2_ (i.e., the reference substance), CFC-11, CFC-114, and CFC-13 have GWPs of 1.00 × 10^0^ kg-CO_2_/kg-CO_2_, 4.00 × 10^3^ kg-CO_2_/kg-CFC-11, 9.30 × 10^3^ kg-CO_2_/kg-CFC-114, and 8.50 × 10^3^ kg-CO_2_/kg-CFC-13, respectively. They can be multiplied by the classification results for the ready-mixed concrete 25-240-15 (4.20 × 10^2^ kg-CO_2_/m^3^, 2.05 × 10^−9^ kg-CFC-11/m^3^, 2.10 × 10^−9^ kg-CFC-114/m^3^, and 4.40 × 10^−10^ kg-CFC-12/m^3^), and added to calculate the impact of this concrete on global warming (4.29 × 10^2^ kg-CO_2_eq/m^3^).

Table 4 lists the LCA results for ready-mixed concrete, produced using electric power with cement, coarse aggregates, fine aggregates, fly ash, and water as the major raw materials. Various emissions and wastes are generated during production.

The LCA results may vary depending on the transport of materials, availability of resources, and improvements in technologies in industry. Therefore, in this study, an environmental impact assessment was conducted using the national LCI DB, designated as a standard in Korea. The national LCI DB comprises the data calculated by setting the standard production process for materials. To assess the environmental impacts of these products, the Ministry of Environment’s LCI Database was utilized. In determining the specialized environmental impacts, the following impact categories were utilized: abiotic depletion, global warming, ozone depletion, photochemical oxidation, acidification, eutrophication, eco-toxicity, and human toxicity. Table 4 presents some of the environmental impacts of the selected construction materials. Ready-mixed concrete and cement exhibit many similar construction constituents. The table lists the environmental impact specialization values for eight impact categories.

### 5.3. Impact Category Normalization/Weighting for Each Construction Material

In order to determine the relative importance of the eight environmental impacts of construction materials, an integrated factor was calculated based upon the weighting factor applied to each impact category. The environmental impact in one impact category was divided by the total environmental impact arising from all impact categories for a certain area over a certain period of time, and weighting (i.e., to represent the relative importance of each impact category) was performed for each category. Table 5 lists the global normalization factor and CML 2001 weighting factor used by the Center of Environmental Science.

Table 6 presents the endpoint coefficients of 22 construction materials covering the seven major materials from the national LCI DB and MOLIT environmental DB. The impact categories were analyzed by applying a cumulative weight cutoff of 95%, which is the impact category exemption standard for each construction material. The cutoff criterion was set to 95% because the exemption standard specified in ISO 14040s was applied as described above. If this value is increased, materials with insignificant impacts on the environment will be included; if decreased, materials with significant impacts on the environment will be excluded, thereby affecting the assessment results.

Normalization/weighting reference values were applied to the specialization results. The top major impact categories for ready-mixed concrete were ADP, GWP, and POCP in descending order, whereas those for cement were GWP and ADP. POCP was strongly influenced only by Portland and blast-furnace slag cement. Paint strongly influenced ADP, GWP, HTP, and PMF (in descending order). Rebar affected ADP, GWP, and AP, whereas glass influenced ADP, GWP, and the freshwater eco-toxicity potential.

ADP and GWP were the most impactful categories for boards, whereas plywood showed the greatest influence on HTP. The chemical treatment process was significantly impacted by the use of a coagulant (PAC).

The values of GWP, which were among the highest in the specialization results for all of the construction materials, became relatively small after normalization since the reference values were large. To propose the specialized impact categories for construction materials, a contribution ≥95% was considered the threshold, as indicated in Table 7. In this case, impact categories of these construction materials that accounted for more than 80% of the weighting factor were selected as common impact categories. Additionally, impact categories of construction materials, excluding the common impact categories, which accounted for more than 95% of the weighting factor, were proposed as specialization impact categories for each of them; GWP and ADP were determined to be common impact categories. The specialization impact categories for concrete were AP, POCP, and HTP, and that for cement was POCP. The specialization impact category for paint was HTP, and that for rebar was AP. The specialization impact categories for glass were AP and HTP, whereas those for boards were EP and AP.

## 6. Discussion

**Figure 3:**
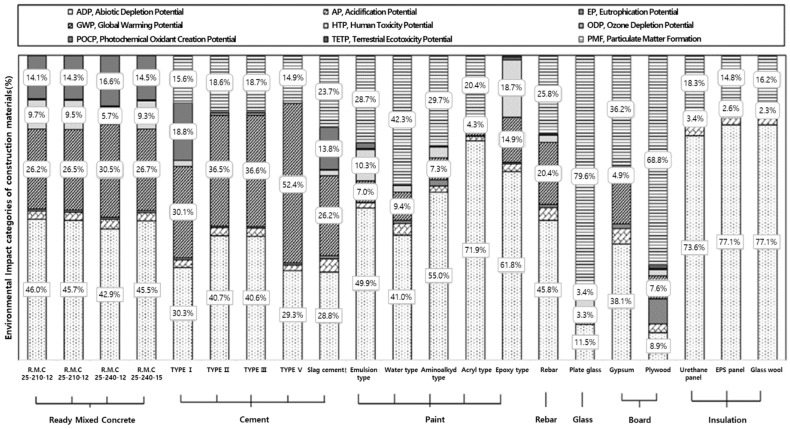
Major environmental impact categories of construction materials.

A detailed analysis of the specialized impact categories of construction materials is presented in Figure 3. According to the results for the impact categories of ready-mixed concrete, the proportions of GWP, ADP, and POCP were greater than 90%. It can be observed that the values of GWP, EP, and POCP tended to increase, whereas the value of ADP tended to decrease in correspondence with the strength rating of the ready-mixed concrete, attributed to the higher quantity of cement and smaller quantity of aggregates (gravel and sand) used to obtain ready-mixed concrete with a higher strength rating. In this instance, GWP, EP, and POCP increased because the quantity of cement, which had a high-environmental impact on these categories, increased; conversely, ADP decreased due to a decrease in aggregates, which have a high environmental impact on ADP.

Accordingly, GWP, ADP, and EP tended to increase in ascending order for Portland cement types 3 (rapid hardening), 5 (sulfate resistant), 1 (ordinary Portland cement (OPC)), and 2 (moderate heat cement). Among the calcium-silicate compounds that constitute cement clinkers, the content of belite (C_2_S, 2CaO·SiO_2_) generally increased, in ascending order for types 3, 5, 1, and 2 Portland cement, whereas that of alite (C_3_S, 3CaO·SiO_2_) decreased. The environmental impact of belite appears to be greater than that of alite based on the GWP, ADP, and EP. As a result, blast-furnace-slag cement has a significantly lower environmental impact on GWP and ADP than Portland cement, while having a significantly higher impact on ODP and POCP. In this study, we demonstrated that blast-furnace slag, used as an additive in blast-furnace slag cement, has a lower environmental impact when compared to OPC and clinker, whereas it demonstrates a higher environmental impact when compared to ODP and POCP. Hence, blast-furnace slag cement is considered favorable with regards to its GWP and ADP; however, its environmental friendliness may depend on the impact categories considered in the life cycle assessment of the building.

In this study, assessment criteria were presented by quantitatively calculating environmental impact factors that must be considered in addition to GWP during LCIA for construction materials. This study is different from previous studies because an assessment method considering of eight environmental impact factors, including resource consumption and human toxicity, was presented instead of the existing environmental impact assessment that simply considered only carbon emissions.

However, the analyzed results do not include all the construction materials as assessment targets. As described above, this study targeted major construction materials. Correspondingly, it is considered necessary to conduct assessments by including more construction materials in future research.

Paints were observed to exert different environmental impacts on each impact category depending on their type. Although the control process determines the extent of waterproofing and coating type, relatively similar environmental impacts were observed for all impact categories within the same product group. The environmental impacts in most of the categories decreased in the order of epoxy, amino-alkyd resin, acrylic resin, emulsion resin, and water-based paint, thereby indicating that the inputs and outputs of most impact substances in the LCI DB, including reference substances, such as CO_2_, PO_4_^3−^, and SO_2_, decreased in the same order, as discussed previously. The major causes of eco-toxicity and human toxicity were observed to be the chemical processes involved in the manufacturing method; the specific impact factors were polycyclic aromatic hydrocarbons (PAH), Barium (Ba), and Vanadium (V). Pollutants, such as NO_x_, Ni, and dust affect the atmosphere, whereas PAH mostly affects aquatic systems.

## 7. Conclusions

This study aimed to select the major impact categories in an LCIA associated with major construction materials. The following conclusions are drawn:

1. A database essentially required for the construction of LCA was presented by selecting seven major construction materials with significant influences on the environment, such as concrete, rebars, and paint, and 22 mainly used construction materials based on the literature survey.
2. Quantitative results were calculated for eight environmental impact categories (GWP, ODP, ADP, AP, EP, POCP, HTP, and TETP) by conducting LCA for the database of the selected construction materials. Based on this, criteria for selecting major environmental impact factors for construction materials were formulated.
3. Criteria for selecting environmental impact factors that must be evaluated for construction materials were prepared using the results, and major environmental impact factors were derived for each material.
4. Global warming and abiotic depletion were derived as common impact categories. The specialized impact categories included acidification and photochemical oxidant creation because of concrete, human toxicity caused by paint, acidification caused by rebars, acidification and human toxicity caused by glass, and eutrophication and acidification caused by boards.
5. The key result of this study was the derivation of environmental impact factors that must be considered for all major construction materials based on life cycle environmental impact assessments. Consequently, factors that must be considered for each material in addition to GWP were distinguished from other factors (Figure 2)
6. In addition to GWP, various other environmental impacts must be considered for improved assessment accuracy in terms of the environmental friendliness of construction materials to facilitate results with improved relevance.

## Figures and Tables

**Table 1:** Life cycle impact assessment methods [38].

Category	Method	Nation	Institute	Data Scope	Environmental Impact Category
midpoint level	CML 2001	Netherlands	Center of Environmental Science of Leiden University	global/Europe	Acidification potential, climate change, eutrophication potential, freshwater aquatic eco-toxicity, human toxicity, marine aquatic eco-toxicity, photochemical oxidation, resources, stratospheric ozone depletion, terrestrial eco-toxicity
	EDIP 2003	Denmark	Technical University of Denmark	Europe	acidification, terrestrial eutrophication, photochemical ozone exposure of plants, photochemical ozone exposure of human beings, global warming
	TRACI	USA	US EPA	North America	ozone depletion, global warming, acidification, eutrophication, photochemical oxidation, eco-toxicity, human health
endpoint level	Eco- indicator 99	Netherlands	PRé Sustainability	global/Europe	mineral and fossil resources, ecosystem quality, human health
	EPS 2000	Sweden	IVL	North America/Europe	life expectancy, severe morbidity, morbidity, severe nuisance, nuisance, crop growth capacity, wood growth capacity

**Table 2:** Life cycle impact (LCI) database (DB) of construction materials.

Category	LCI DB			
	No.	Korea LCI DB	National DB for Environmental Information of Building Products	Functional Unit
ready-mixed concrete	1	ready-mixed concrete 25-210-12	·	m^3^
	2	ready-mixed concrete 25-210-15		
	3	ready-mixed concrete 25-240-12		
	4	ready-mixed concrete 25-240-15		
rebar	5	electric steel deformed bars	·	kg
			·	
paint	6	paint–emulsion type	·	kg
	7	paint–water type	·	kg
	8	paint–amino alkyd type	·	kg
	9	paint–acryl type	·	kg
	10	paint–epoxy type	·	kg
glass	11	plate glass	·	kg
cement	12	cement	·	kg
	13	Portland cement type I		kg
	14	Portland cement type II	·	kg
	15	Portland cement type III	·	kg
	16	Portland cement type V	·	kg
	17	Blast furnace slag cement	·	kg
insulation	18	·	urethane panel	kg
	19		EPS panel	kg
	20		glass wool	kg
board	21	gypsum board		kg
	22	·	plywood board	kg

**Table 3:** Classification of building materials in the LCI DB [16] (CO_2_: carbon dioxide, CFC: chlorofluorocarbons).

Classification	Environment	Ready-Mixed Concrete 25-240-15	Electric Steel Deformed Bars	Paint–Water Type
CO_2_ [kg-CO_2_/m^3^]	air	4.20 × 10^2^	3.40 × 10^−1^	1.07 × 10^3^
CFC-11 [kg-CFC-11/m^3^]	air	2.05 × 10^−9^	4.02 × 10^−13^	6.04 × 10^−7^
CFC-114 [kg-CFC-114/m^3^]	air	2.10 × 10^−9^	4.12 × 10^−13^	6.18 × 10^−7^
CFC-12 [kg-CFC-12/m^3^]	air	4.40 × 10^−10^	8.64 × 10^−14^	1.30 × 10^−7^
ethane [kg-Ethane/m^3^]	air	1.91 × 10^−3^	4.34 × 10^−7^	5.92 × 10^−3^
ethanol [kg-Ethanol/m^3^]	air	2.73 × 10^−6^	6.19 × 10^−10^	7.88 × 10^−6^
Halon-1301 [kg-Halon-1301/m^3^]	air	3.82 × 10^−6^	8.68 × 10^−10^	2.15 × 10^−6^
HCI [kg-HCI/m^3^]	air	1.49 × 10^−4^	2.18 × 10^−7^	5.76 × 10^−2^
HF [kg-HF/m^3^]	air	1.01 × 10^−5^	6.18 × 10^−9^	1.83 × 10^−3^
NO_2_ [kg-NO_2_/m^3^]	air	6.93 × 10^−4^	1.38 × 10^−6^	2.50 × 10^−3^
SO_2_ [kg-SO_2_/m^3^]	air	2.67 × 10^−1^	4.42 × 10^−4^	3.63 × 10^0^
PO_4_^3−^ [kg-PO_4_^3^/m^3^]	water	1.76 × 10^−4^	4.22 × 10^−8^	5.74 × 10^−2^
crude oil [kg-Crude oil/m^3^]	soil	4.61 × 10^1^	2.35 × 10^−2^	2.76 × 10^2^
lead (Pb) [kg-Lead (Pb)/m^3^]	soil	1.39 × 10^−6^	2.89 × 10^−15^	1.08 × 10^−3^

**Table 4:** Environmental impact coefficients of construction materials [16].

Category	Construction Materials	DB ^1^	Functional Unit	Environmental Impact Categories							
				GWP	ADP	EP	ODP	POCP	AP	HTP	PMF
				kg-CO_2-eq_	kg	kg-PO_4_^3−^_-eq_	kg-CFC_-eq_	kg-C_2_H_4-eq_	kg-SO_2-eq_	kg DCB_-eq_	kg DCB_-eq_
ready-mixed concrete	ready-mixed concrete 25-21-12	A	m^3^	4.09 × 10^2^	6.81 × 10^−1^	7.96 × 10^−2^	4.65 × 10^−5^	1.02 × 10^0^	1.55 × 10^0^	2.10 × 10^−18^	2.78 × 10^−19^
	ready-mixed concrete 25-21-15	A	m^3^	4.19 × 10^2^	6.94 × 10^−1^	8.08 × 10^−2^	4.61 × 10^−5^	1.13 × 10^0^	1.56 × 10^0^	2.19 × 10^−18^	2.82 × 10^−19^
	ready-mixed concrete 25-24-12	A	m^3^	4.14 × 10^2^	6.79 × 10^−1^	8.12 × 10^−2^	4.34 × 10^−5^	1.07 × 10^0^	1.96 × 10^0^	2.15 × 10^−18^	2.65 × 10^−19^
	ready-mixed concrete 25-24\-15	A	m^3^	4.29 × 10^2^	7.05 × 10^−1^	8.20 × 10^−2^	4.59 × 10^−5^	1.15 × 10^0^	2.08 × 10^0^	2.22 × 10^−18^	3.08 × 10^−19^
cement	cement	A	kg	1.06 × 10^0^	1.13 × 10^−3^	1.79 × 10^−4^	3.55 × 10^−8^	3.03 × 10^−4^	2.79 × 10^−3^	1.17 × 10^−18^	5.58 × 10^−19^
	Portland cement type I	A	kg	9.48 × 10^−1^	7.36 × 10^−4^	1.17 × 10^−4^	1.70 × 10^−8^	2.60 × 10^−3^	1.12 × 10^−3^	2.74 × 10^−19^	2.64 × 10^−19^
	Portland cement type II	A	kg	9.49 × 10^−1^	1.52 × 10^−3^	1.16 × 10^−4^	1.39 × 10^−9^	1.74 × 10^−4^	1.12 × 10^−3^	1.33 × 10^−19^	1.35 × 10^−19^
	Portland cement type III	A	kg	9.36 × 10^−1^	6.58 × 10^−4^	1.15 × 10^−4^	1.25 × 10^−9^	1.66 × 10^−4^	1.11 × 10^−3^	6.50 × 10^−17^	6.55 × 10^−17^
	Portland cement type V	A	kg	9.43 × 10^−1^	6.64 × 10^−4^	9.53 × 10^−5^	1.28 × 10^−9^	1.42 × 10^−4^	6.49 × 10^−3^	1.13 × 10^−19^	1.11 × 10^−19^
	blast furnace slag cement	A	kg	2.05 × 10^−1^	1.91 × 10^−4^	9.99 × 10^−4^	4.14 × 10^−9^	5.00 × 10^−4^	1.48 × 10^−2^	1.27 × 10^−19^	1.20 × 10^−19^

^1^ A: Korea LCI DB; B: National DB for environmental information of building products.

**Table 5:** Normalization and weighting factors of impact categories [16].

Impact Category	Normalization Factor		Weighting Factor
	Value	Unit	Value
abiotic depletion potential (ADP)	2.49 × 10^4^	g/person-year	2.31 × 10^−1^
global warming potential (GWP)	5.53 × 10^6^	g CO_2-eq_/person-year	2.88 × 10^−1^
ozone depletion potential (ODP)	4.07 × 10^1^	g CFC_-eq_/person-year	2.92 × 10^−1^
photochemical oxidant creation potential (POCP)	1.03 × 10^4^	g C_2_H_4-eq_/person-year	6.50 × 10^−2^
acidification potential (AP)	3.98 × 10^4^	g SO_2-eq_/person-year	3.60 × 10^−2^
eutrophication potential (EP)	1.31 × 10^4^	g PO_4_^3−^_-eq_/person-year	3.80 × 10^−2^
particulate matter formation (PMF)	1.63 × 10^3^	g PM_10-eq_/person-year	2.16 × 10^−1^
HTP	1.48 × 10^6^	g DCB_-eq_/person-year	1.05 × 10^−1^

**Table 6:** Endpoint coefficients corresponding to the environmental impact categories of construction materials [16].

Category	Construction Material	DB ^1^	Functional Unit	Environmental Impact Categories (%)							
				GWP	ADP	EP	ODP	POCP	AP	HTP	PMF
ready-mixed concrete	ready-mixed concrete 25-210-12	A	m^3^	39.06	41.26	0.31	0.36	8.42	1.41	2.31	0.03
	ready-mixed concrete 25-210-15	A	m^3^	39.44	40.97	0.31	0.35	8.55	1.38	2.26	0.03
	ready-mixed concrete 25-240-12	A	m^3^	44.89	38.06	0.36	0.21	9.80	1.14	1.34	0.02
	ready-mixed concrete 25-240-15	A	m^3^	39.74	38.77	0.31	0.35	8.65	1.36	2.21	0.03
cement	cement	A	kg	63.25	30.47	0.46	0.17	0.18	0.21	0.94	0.01
	Portland cement type I	A	kg	52.00	31.44	0.30	0.08	13.02	1.09	0.52	0.01
	Portland cement type II	A	kg	58.70	39.43	0.24	0.01	0.20	1.07	0.12	0.00
	Portland cement type III	A	kg	58.89	39.22	0.25	0.01	0.19	1.06	0.11	0.00
	Portland cement type V	A	kg	73.80	24.82	0.30	0.01	0.03	0.63	0.14	0.00
	blast furnace slag cement	A	kg	50.70	33.48	0.67	0.08	10.73	2.07	0.56	0.01
paint	paint–emulsion type	A	kg	22.63	60.29	0.21	0.60	0.27	0.87	4.47	0.07
	paint–water type	A	kg	15.99	68.13	0.19	0.34	1.71	0.86	4.26	0.06
	paint–amino alkyd type	A	kg	25.92	68.21	0.90	0.05	0.48	2.56	0.38	0.01
	paint–acryl type	A	kg	25.92	68.21	0.90	0.05	0.48	2.56	0.38	0.01
	paint–epoxy type	A	kg	8.74	88.64	0.21	0.03	0.16	0.73	0.61	0.00
rebar	electric steel deformed bars	A	kg	40.14	54.24	0.68	0.08	0.26	1.99	0.86	0.01
glass	plate glass	A	kg	24.78	52.39	0.51	0.78	0.73	2.66	*5.54*	0.06
board	gypsum board	A	kg	32.76	54.78	1.30	0.27	0.25	2.87	1.14	0.03
	plywood board	B	kg	28.64	20.24	*10.47*	0.10	0.57	2.71	32.64	0.88
insulation	urethane panel	B	kg	6.87	88.08	0.33	0.00	0.43	1.68	1.96	0.00
	EPS panel	B	kg	5.10	91.82	0.28	0.01	0.65	1.55	0.28	0.00
	glass wool	B	kg	4.57	92.66	0.29	0.01	0.42	1.58	0.26	0.00

^1^ A: Korea LCI DB; B: National DB for environmental information of building products.

**Table 7:** Deduction of the major environmental impact categories.

Material		Environmental Impact Categories ^1^						
	GWP	ADP	EP	ODP	POCP	AP	HTP	PMF
concrete	●	●	-	-	○	○	○	-
cement	●	●	-	-	○	-	-	-
paint	●	●	-	-	-	-	○	-
rebar	●	●	-	-	-	○	-	-
glass	●	●	-	-	-	○	○	-
board	●	●	○	-	-	○	-	-
insulation	●	●	-	-	-	-	-	-

^1^ ● Mandatory environmental impact category, ○ Specialized environmental impact category.

## Data Availability

Not applicable.
